# Multi-scenario investment forecast of new energy projects based on multiple linear regression and comprehensive evaluation model of differentiated project priorities

**DOI:** 10.1016/j.heliyon.2023.e23771

**Published:** 2023-12-18

**Authors:** Guang Tian, Xiangyu Chen, Chunsheng Chen, Yang Yang, Jialin Li, Yangyi Zhang

**Affiliations:** a Development Research Center, State Grid Hebei Electric Power Co., Ltd., Shijiazhuang, 050000, China; b State Grid Hebei Electric Power Co., Ltd., Shijiazhuang, 050000, China; c Institute of Economic and Technological Research, State Grid Hebei Electric Power Co., Ltd., Shijiazhuang, 050000, China; d School of Economics and Management, North China Electric Power University, Beijing, 102206, China; e School of Economics and Management, Beijing University of Technology, Beijing, 100124, China

**Keywords:** Clean energy project, Investment prediction model, Priority comprehensive evaluation method, Multi-scene simulation

## Abstract

As China's resource shortage and environmental pollution intensify, the demand for new energy and electric energy substitution is becoming higher and higher. Accurately predicting the investment scale of China's new energy projects is of great practical significance for improving the efficiency of resource allocation and economically meeting energy demand. This paper builds a scientific and precise investment model for new energy projects from both macro and micro perspectives. First, from a macro perspective, considering macro indicators such as the external environment and internal economy, an annual total investment forecast model based on multiple linear regression is constructed, in order to predict the annual total investment scale of new energy investment entities and achieve preliminary accurate investment; second, designed the evaluation index system of different project priorities from three perspectives of external environment, internal development of enterprises and social development, and constructed the comprehensive weight design method based on AN-EWM and the comprehensive evaluation method of TOPSIS, in order to realize the priority of differentiated projects. Sorting; finally, a new energy project located in a city in northern China is selected as the research subject, and a multi-scenario example analysis is carried out. The results show that the new energy project investment scale index system constructed in this paper can effectively evaluate the investment capacity of the main body of the new energy project, and can better predict the total investment of the new energy investment project, so that the deviation rate can be controlled within 5 %, and the priority evaluation model constructed in this paper can provide a complete calculation method and a reference method for the judgement of the investment priority, which can promote accurate investment.

## 1 Introduction

In recent years, problems such as energy crisis and frequent occurrence of extreme climate have been intensifying worldwide. For China, the relatively large population determines that the problem is even more serious. In order to ensure energy security and cope with the climate crisis, all countries are actively promoting energy reform and transformation, and the Chinese government pays particular attention to this issue by continuously increasing the development of clean energy to reduce its dependence on fossil energy and carbon emissions [1]. In this context, the construction and development of clean energy projects in China has been vigorously pushed forward, and investment forecasts for new energy projects are related to the projects themselves and the end-users' immediate interests and affects the efficiency of resource allocation. Therefore, it is of great practical significance to study how to formulate a scientific and reasonable investment forecasting forecast model and a differentiated project investment priority model to avoid wasting investment resources and meet the growing energy demand in a clean way.

At present, domestic and foreign researches have carried out a series of studies on investment scale prediction. Relevant scholars have done some research on the forecast of power grid investment. Wang et al. [2] first analyzed the main influencing factors of power grid company investment, and then used the support vector machine model to predict and analyze the investment scale of a certain city power grid company under different boundary conditions, and finally gave corresponding conclusions and suggestions. Liu et al. [3] proposed a power grid investment portfolio forecasting model based on gray prediction, BP neural network, and multiple regression, and used the proposed combination forecasting model to predict power grid investment in a certain area. Xie et al. [4] first explored the indicator system of external driving factors of power grid investment, from the four aspects of power demand factors, grid security factors, energy transition factors and technological innovation factors, combined with gray correlation theory, screening key driving factors, and constructing provincial power grids. Long-term investment scale prediction model; finally, through the support vector machine algorithm, the investment scale of the provincial power grid from 2019 to 2022 is predicted. Mo and Ye [5] took the province A power grid company as an example, and proposed a power grid investment prediction model based on the asset wall theory. Ji et al. [6] studied the influencing factors of power grid investment from the perspectives of macroeconomics and power market, and constructed a power grid investment prediction model based on the ARMA model.

In the evolving landscape of power grid investment prediction, recent studies have started to integrate the complexities of renewable energy adoption. For instance, the variability of renewables necessitates novel approaches in predictive modeling to ensure grid stability and meet investment requirements. Researchers like Zhou et al. [7] have examined the impact of renewable energy integration on power grid investments, leveraging advanced statistical methods to predict the necessary scale of investment with higher accuracy. Technological advancements in predictive analytics are also being adopted, as indicated by Smith and Zhao [8], who applied machine learning techniques to forecast grid investment needs, outperforming traditional statistical models. This signifies a trend towards more sophisticated, data-driven approaches in the field. Furthermore, the validation of these models is crucial. Case studies, such as those presented by Kumar and Singh [9], compare the predicted investment scales with actual outcomes, providing an empirical basis for the models' effectiveness. These studies often highlight the need for high-quality, reliable data, emphasizing the role of big data analytics in future predictive models, as discussed by Lee and Park [10]. Additionally, regulatory frameworks have significant implications for investment predictions. The work of Gomez and Tran [11] illustrates how changes in policy can be factored into predictive models to better align with the new round of transmission and distribution price supervision cycles. Lastly, the integration of financial risk assessment within predictive models is an emerging focus area. The research by Fernandez and Patel [12] underscores how power grid companies can use predictive models to manage financial risks associated with large-scale investments.

The above literatures have laid a certain research foundation for the research of this paper, but there are few prediction models about the investment scale of new energy projects, and the current research only studies the total investment scale from a macro perspective, without predicting the total investment scale. Build the investment priority model of differentiated projects from a micro perspective. Therefore, this paper takes the investment of new energy projects as the main research body, combines macro and micro, from a macro perspective, on the basis of constructing the total annual investment scale model, from a micro perspective, constructs a new energy based on the comprehensive evaluation method of AN-EWM-TOPSIS The project priority evaluation model realizes the prediction of total macro investment and the prioritization of micro investment, which is of great practical significance for guiding the economic sustainable investment development of new energy projects.

## 2 Construction of investment prediction evaluation index of clean energy project

When investment entities invest in new energy projects, they usually need to comprehensively consider internal and external macro-influencing factors in order to predict the total investment scale. Therefore, from a macro perspective, this section builds a forecast model for the investment scale of new energy project investors to predict the total annual investment scale.

### 2.1 Analysis of influencing factors of investment scale

The investment in clean energy projects is affected by the external environment and the internal environment of the investor.

From the external environment, clean energy projects serve electric power. As one of the most important energy sources, electric power is a necessity for social production and people's life. The investment of the investor in clean energy projects will be affected by factors such as resident consumption index and load demand. Among them, the resident consumption index reflects the overall resident consumption capacity of the region, and the load demand directly reflects the changing trend of power demand.

From the perspective of internal environment, the operation of enterprises also affects the proportion of resource input to a large extent. For example, the asset liability ratio, operating income and profit of an enterprise will directly affect the investment capacity of the investor and the total investment scale.

Therefore, combined with the above analysis, the above factors are classified into two categories: external factors and internal factors. Among them, external factors include regional consumer price index and load demand; Internal factors include asset liability ratio, operating income and total profit.

### 2.2 Construction of investment scale model

#### 2.2.1 Investment scale prediction model

Regression analysis method refers to an analysis method that uses the principle of data statistics to mathematically process a large number of statistical data, determine the correlation between dependent variables and some independent variables, establish a regression equation (function expression) with good correlation, and extrapolate it to predict the change of dependent variables in the future [13].

Construct the quantitative relationship between annual total investment and variables:

(1) $$Y_{j}=a_{i}m_{i}+b$$

In equation (1), $Y_{j}$ is the historical actual total input of n-th years, $m_{i}$ is the i-th influencing factor, $a_{i}$ is the variable after regression analysis, and b is the deviation.

#### 2.2.2 Investment scale cross test model

On the basis of solving the total investment scale model, in order to verify the accuracy of the model, the established regression mathematical model is tested by using the estimation history cross bias test method.

In the given total input data over the years, most of the samples are used to build the model, and a small part of the samples are left to be tested with the just established model, and the measurement deviation of this small part of the samples is calculated, so as to ensure the effectiveness and popularization of the method and model.

Take the historical actual total input $Y_{j}\left(j=1,2,\ldots ,n\right)$ of n years for cross test, and use the total input of n-1 year and relevant index data to predict the total input of the remaining years ${Y_{j}}^{\prime }\left(j=1,2,\ldots ,n\right)$, and then calculate the deviation from the actual input $\sigma_{j}$, Among them, $\sigma_{j}=\left(Y_{j}-{Y_{j}}^{\prime }\right)/Y_{j}$. The details are as follows in Table 1:

**Table 1:** Cross inspection results and deviations.

year	Predicted value	Actual value	Deviation
D	${Y_{1}}^{\prime }$	$Y_{1}$	$\sigma_{1}$
2	${Y_{2}}^{\prime }$	$Y_{2}$	$\sigma_{2}$
… …	… …	… …	… …
n	${Y_{n}}^{\prime }$	$Y_{n}$	$\sigma_{n}$

## 3 Comprehensive evaluation model for investment priority of differentiated new energy projects

On the basis of forecasting the total annual investment of new energy projects from a macro perspective, with the continuous development of new energy technologies, new energy projects are gradually increasing, and rational arrangement of internal investment priorities is conducive to promoting external economic and environmental development and meeting user needs. It is of great significance to improve the internal economy and sustainable development. Therefore, this section further builds a comprehensive evaluation model of new energy project investment priorities from a microscopic perspective to achieve scientific ranking of project investment priorities.

### 3.1 New energy project investment priority comprehensive evaluation index system

This section constructs a comprehensive evaluation index system of priorities from three aspects: external environment, improvement of profitability and social contribution. Among them, the main evaluation of the external environment is the support of the external environment for different new energy projects and the market development prospects. The main evaluation of the profitability of new energy projects is the profitability of new energy projects and the current technical support capabilities, whether to carry out new technology and new resources. Further investment, and social contribution mainly evaluates the impact of different new energy projects on the social economy, ecological environment and the public. The specific evaluation index system is shown in the following Table 2.

**Table 2:** New energy project priority comprehensive evaluation index system.

Level I index	Level II index	Level III index
External environment (A)	Market prospect (A1)	Electricity occupancy (A11)
		Population coverage (A12)
		Improve power reliability (A13)
		Power quality satisfaction (A14)
		Growth rate of new users (A15)
	Macro policy (A2)	Policy support (A21)
		Policy restrictions (A22)
Improve profitability (B)	Project investment (B1)	Net income to equity ratio (B11)
		NPV index (B12)
		Internal rate of return (B13)
	Project output (B2)	Return on investment (B21)
		Static investment payback perio (B22)
	Technical support (B3)	Technical maturity (B31)
		innovation ability (B32)
Social contribution (C)	Impact on socialeconomy (C1)	Promote the development of industrial economy (C11)
		Promote regional economic development (C12)
		Benefits of technological progre (C13)
		Employment benefits (C14)
	Impact on ecological environment (C2)	energy conservation (C21)
		Social resource sharing (C22)
		Reduce environmental pollution (C23)
	User satisfaction (C3)	Service convenience (C31)
		Project green index (C32)

### 3.2 New energy project priority index weight design model based on NA-EWM

In order to fully reflect the objectivity of weight determination and reduce the influence of human factors, this paper adopts a comprehensive weight determination method combining network analysis (NA) and entropy weight method (EWM) [14,15] to ensure the rationality of weight to the greatest extent.

#### 3.2.1 Model of entropy weight method

Step 1 Collect and process the original data of indexes. Thus, the standardized index recto ($Y_{ij}$) is obtained.

(2) $$Y_{ij}=\left[\begin{array}{cccc} y_{11} & y_{12} & ... & y_{1m}\\ y_{21} & y_{22} & ... & y_{2m}\\ ... & ... & ... & ...\\ y_{n1} & y_{n2} & ... & y_{nm} \end{array}\right]$$

In equation (2), $y_{ij}$ represents the standardized value of the *i* index under the *j* level.

Step 2 The uncertainty value of the index ($H\left(y_{i}\right)$) is obtained by equation (3):

(3) $$H\left(y_{i}\right)=-\sum_{j=1}^{n}\left(\frac{1+y_{ij}}{y_{i}=\sum_{j=1}^{n}\left(1+y_{ij}\right)}\ln\frac{1+y_{ij}}{y_{i}=\sum_{j=1}^{n}\left(1+y_{ij}\right)}\right)$$

Step 3 Calculate the information entropy of the index ($e\left(y_{i}\right)$):

(4) $$e\left(y_{i}\right)=\frac{H\left(y_{i}\right)}{\ln\;n}$$

In equation (4), $0\leq e\left(y_{i}\right)\leq 1$.

Step 4 Calculate the objective weight of the index ($\xi_{i}$):

(5) $$\xi_{i}=\frac{1-e\left(y_{i}\right)}{m-\sum_{i=1}^{m}e\left(y_{i}\right)}$$

In equation (5), $0\leq \xi_{i}\leq 1$, $\sum_{i=1}^{m}\xi_{i}=1$.

#### 3.2.2 Model of network analysis

The ranking vector uses the characteristic root through the network analysis model and combines all the ranking vectors of the network elements into a matrix:

(6) $$W_{ij}=\left[\begin{array}{cccc} w_{i1}^{j1} & w_{i1}^{j2} & \cdots & w_{i1}^{jn_{j}}\\ w_{i2}^{j1} & w_{i2}^{j2} & \cdots & w_{i2}^{jn_{j}}\\ \cdots & \cdots & \cdots & \cdots \\ w_{in_{j}}^{j1} & w_{in_{j}}^{j2} & \cdots & w_{in_{j}}^{jn_{j}} \end{array}\right]$$

In equation (6), $W_{ij}$ is a column vector representing the importance ranking vector of $C_{i}$ to $C_{j}$. Further, combining the ordering vectors of the interactions of all network layer elements, a super matrix under the control element is obtained as equation (7):

(7) $$W=\begin{array}{c} 1\\ \cdots \\ n_{1}\\ 1\\ \cdots \\ n_{2}\\ \ldots \\ 1\\ \ldots \\ n_{N} \end{array}\left[\begin{array}{cccc} W_{11} & W_{12} & \cdots & W_{1N}\\ W_{21} & W_{22} & \cdots & W_{2N}\\ W_{N1} & W_{N2} & & W_{N3} \end{array}\right]$$

And then calculate the limit hypermatrix.

(8) $$\underset{k\to \infty }{\lim\left(1/N\right)}\sum_{k=1}^{N}{\bar{W}}^{k}$$

In equation (8), $\bar{W}={\left(\bar{W}\right)}_{n\times n}$, $a_{ij}$ (i,j=1,2,⋯,N) is the weighting factors. If the above limit is convergent and unique, the value of the corresponding row of the meta-matrix is the stable weight of each evaluation index.

#### 3.2.3 Model of comprehensive weight model based on AN-EWM

In order to effectively obtain the advantages of the above weight determination model, this section uses the linear weighting method to design a comprehensive weight model to realize the scientific determination of the weight of the evaluation index system and lay the foundation for determining the priority of new energy projects. The model is:

(9) $$\gamma_{i}=\alpha \omega_{i}+\left(1-\alpha \right)\xi_{i}$$

In equation (9), $\omega_{i}$ represents the AN weight; $\xi_{i}$ represents the WEM weight; $\gamma_{i}$ represents the Comprehensive weight; And $\sum \omega_{i}=1$, $\sum \xi_{i}=1$, $\sum \gamma_{i}=1$, 0≤α≤1.

### 3.3 New energy project priority comprehensive evaluation model based on TOPSIS

#### 3.3.1 Index standardization processing model

As for decision-making indexes, they can be classified by changing methods. There are three common classification methods, namely, forward indexes, reverse indexes and moderate indexes [16].

(1) Positive indexes

The index standardization model is:

(10) $$y=\frac{x-x^{\min}}{x^{\max}-x^{\min}}$$

In equation (10), $x^{\max}$ is the maximum value expected to be achieved, $x^{\min}$ is the historical minimum.

(2) Reverse index

The index standardization model is:

(11) $$y=\frac{x^{\max}-x}{x^{\max}-x^{\min}}$$

In equation (11), $x^{\max}$ is the historical maximum, $x^{\min}$ is the minimum value expected to be achieved.

(3) Moderate index

According to model (10), the moderate index is transformed into the reverse index.

(12) $$x^{\prime }=\left|x-x^{mid}\right|$$

In equation (12), $x^{mid}$ is the expected moderate value.

#### 3.3.2 Comprehensive evaluation model based on TOPSIS

Due to the strong comparability of objective data of various indicators between different new energy project businesses, when quantifying the investment value of different new energy project businesses, the distance between superior and inferior solutions (TOPSIS) method can be selected. The principle of this method is: if an indicator of a scheme is closer to the maximum value of the indicator in all schemes, and farther away from the minimum value of the indicator, the score of the indicator will be higher; the score of each indicator of the scheme is multiplied by the weight That is, the comprehensive score is obtained, and the comprehensive score is used to compare the pros and cons of the scheme. Specific steps are as follows [17,18].

Step 1 Obtain the original matrix ($P_{mn}$) according to the data of the evaluation index, then use the maximum value minus the very small index to realize the positive speech, and finally perform normalization processing to obtain the change matrix ($P_{mn}^{\prime }$) in equation (13).

(13) $${P_{ij}}^{\prime }=\frac{P_{ij}}{\sqrt{\sum_{i=1}^{m}{P_{ij}}^{2}}}$$

Step 2 Based on the weight coefficient ($\omega_{j}$), Weighting the normalized data to form a weighted normalization matrix is shown in equations (14), (15):

(14) $$V={\left(\omega_{j}P_{ij}\right)}_{mn}$$

(15) $$V={\left(v_{ij}\right)}_{mn}=\left[\begin{array}{cccc} \omega_{1}P_{11} & \omega_{1}P_{21} & ... & \omega_{1}P_{m1}\\ \omega_{2}P_{12} & \omega_{2}P_{22} & ... & \omega_{2}P_{m2}\\ ... & ... & ... & ...\\ \omega_{n}P_{1n} & \omega_{n}P_{2n} & ... & \omega_{n}P_{mn} \end{array}\right]$$

Step 3 Defining a positive ideal scenario ($V^{+}$)and negative ideal scenario ($V^{-}$)

(16) $$V^{+}=\left\{v_{1}^{+},v_{2}^{+},...,v_{n}^{+}\right\}=\left\{\left(\max_{i}v_{ij}|j\in J_{1}\right),\left(\min_{i}v_{ij}|j\in J_{2}\right)|i=1,2,...,m\right\}$$

(17) $$V^{-}=\left\{v_{1}^{-},v_{2}^{-},...,v_{n}^{-}\right\}=\left\{\left(\min_{i}v_{ij}|j\in J_{1}\right),\left(\max_{i}v_{ij}|j\in J_{2}\right)|i=1,2,...,m\right\}$$

In equations (16), (17), $J_{1}$ represents the set of benefit indicators, and $J_{2}$ represents the set of cost indicators.

Step 4 Calculate the Euclidean distance

Assuming that the distance from the scheme i(i=1,2,...,n) to the positive ideal scheme is $S_{i}^{+}$, and the distance to the negative ideal scheme is $S_{i}^{-}$, then in equations (18), (19)

(18) $$S_{i}^{+}=\sqrt{\sum_{j=1}^{n}{\left(v_{ij}-v_{j}^{+}\right)}^{2}}$$

(19) $$S_{i}^{-}=\sqrt{\sum_{j=1}^{n}{\left(v_{ij}-v_{j}^{-}\right)}^{2}}$$

Step 5 Calculate the relative closeness

The closeness of the scheme i(i=1,2,...,n) to the ideal solution is equation (20):

(20) $$e_{i}=\frac{S_{i}^{-}}{S_{i}^{+}+S_{i}^{-}}$$

Therefore, the TOPSIS evaluation value of each scheme is calculated based on the above formula, and the evaluation objects are sorted and selected according to the evaluation value.

## 4 Example analysis

This paper selects a new energy investment entity in northern China for case analysis and research. The main business of this new energy investment company includes comprehensive energy business (coal-to-electricity business), electric vehicle business, distributed energy business, and energy information service business. The software used in this article is Matlab 2023a.

### 4.1 Results of the total annual investment scale of new energy projects

#### 4.1.1 Total investment scale and deviation calculation of new energy projects

Combined with the actual development data of the new energy project investors, based on the analysis of the influencing factors of the total investment scale, the data of the indicators from 2015 to 2020 were obtained through field research and data cleaning (including deletion of abnormal years). As shown in Table 3.

**Table 3:** Historical data of total investment scale and influencing factors.

Year	Annual total input	Consumer price index,	Load demand	Asset liability ratio	Total profit	Operating income
	(10,000 yuan)		(10,000 KWH)	(%)	(10,000 yuan)	(10,000 yuan)
2015	111.4	102.6	1320.94	64.15	7.07	744.14
2016	112.68	102.6	1351.02	64.69	9.02	727.69
2017	106.88	102	1239.62	64.4	8.81	660.71
2018	102.92	101.4	1218	64.19	6.74	641.56
2019	108.98	103.4	1267.41	64.88	6.18	675.99
2020	111.47	102.5	1285	65.21	5	690.25

In combination with Table 3, regression analysis is conducted by using the company's comprehensive plan data from 2015 to 2020, and the quantitative relationship between annual total investment and variables is constructed as follows:

$$Annual\;total\;investment=\left\{ \begin{array}{l} 0.014\;*\;consumer\;price\;index\\ +0.075\;*\;load\;demand\;\left(10000\;KWH\right)\\ +0.079\;*\;total\;profit\;\left(10000\;yuan\right)\\ +0.048\;*\;operating\;income\;\left(10000\;yuan\right)\\ -0.355\;*\;asset\;liability\;ratio \end{array}\right.$$

Based on the analytical formula of the quantitative relationship between the annual total investment and variables, in order to further verify that the regression model has a certain applicability in predicting investment scale, this section uses the cross-check method to test the deviation. The cross-check results are shown in Table 4.

**Table 4:** Cross inspection results and deviations of the historical total input.

Year	Estimate (10,000 yuan)	Actual (10,000 yuan)	Deviation (10,000 yuan)	Deviation rate
2015	114.0109	114.0109	2.6109	2.29 %
2016	115.43965	115.43965	2.75965	2.39 %
2017	103.94757	103.94757	−2.93243	2.82 %
2018	101.30949	101.30949	−1.61051	1.59 %
2019	106.40669	106.40669	−2.57331	2.42 %
2020	108.18745	108.18745	−3.28255	3.03 %

After using the above formula to test the data from 2015 to 2020, the results are shown in Table 4, and the deviation is basically within 5 %. Therefore, to a certain extent, the investment scale can be predicted by the above model.

#### 4.1.2 Aggregate size sensitivity analysis based on key variables

In order to further analyze the impact of changes in different factors on the total investment scale, factors with high correlation coefficients such as regional load demand, total profit, operating income, and asset-liability ratio are selected as the change factors. The data in 2020 is based on sensitivity analysis, to explore the degree of change in the company's total annual investment under different change scenarios. Specific information can be found in Table 5, Table 6, Table 7, Table 8.

**Table 5:** Total input change under load demand change.

Variation degree of load demand	Load demand (10,000 KWH)	Rate of change	Current total investment (10,000 yuan)
−20 %	1028	−21.68 %	88.91245
−10 %	1156.5	−9.78 %	98.54995
10 %	1413.5	8.18 %	117.825
20 %	1542	15.12 %	127.4625
30 %	1670.5	21.09 %	137.1

**Table 6:** Total input change under total profit change.

Change degree of total profit;	Total profit (10,000 yuan)	Rate of change	Current total investment (10,000 yuan)
−20 %	4	−0.07 %	108.1085
−10 %	4.5	−0.04 %	108.148
10 %	5.5	0.04 %	108.227
20 %	6	0.07 %	108.2665
30 %	6.5	0.11 %	108.306

**Table 7:** Total input change under operating income change.

Change degree of operating income	operating income (10,000 yuan)	Rate of change	Current total investment (10,000 yuan)
−20 %	552.2	−6.52 %	101.5611
−10 %	621.225	−3.16 %	104.8743
10 %	759.275	2.97 %	111.5007
20 %	828.3	5.77 %	114.8139
30 %	897.325	8.41 %	118.1271

**Table 8:** Total input change under asset liability ratio change.

Changes in asset liability ratio	Asset liability ratio (%)	Rate of change	Current total investment (10,000 yuan)
−20 %	52.168	4.10 %	112.8174
−10 %	58.689	2.09 %	110.5024
10 %	71.731	−2.19 %	105.8725
20 %	78.252	−4.47 %	103.5575
30 %	84.773	−6.86 %	101.2426

Combining the above tables, it can be seen that the change of load demand has the greatest impact on the total investment scale. When the load demand drops by 20 %, the total investment drops by 21.68 % compared with the original investment, indicating that the user-side demand has a greater impact on the investment scale. Second, the indicator that has a greater impact on the scale of the total investment is the operating cost, indicating that the level of revenue brought by the project has a greater impact on the total investment scale. In the follow-up investment, we should focus on the user-side load demand and revenue level.

#### 4.1.3 Calculation of investment scale considering changes in uncertain factors

Further, in the development of new energy projects, the factors affecting the investment scale are also constantly changing and developing. In order to better predict the company's investment scale, this paper further introduces the use of scenario analysis to measure and consider the new energy under the development scenario of uncertainty. The scale of investment in energy projects. Considering that there are many factors affecting the total investment, and 4.1.2 has carried out sensitivity analysis on some factors, in order to simplify the calculation, this section selects the household consumption index and total profit to re-fit the total investment forecast model. A multi-scenario simulation scheme is further constructed, and the multi-scenario simulation scheme is shown in the following Table 9. And Among them, the average is the average accumulated over several years.

**Table 9:** Total investment forecast scenario setting under multi-scenario growth.

Scene	Predicted Residential Consumption Index (10,000 yuan)	Predicted Total profit (10,000 yuan)	Predicted total investment $Y_{j}$ (10,000 yuan)	Forecast total input change $Y_{j}^{\prime }$ (10,000 yuan)	Forecast total investment growth rate α	Actual total investment (10,000 yuan)	Deviation
A	1.07 average value	1.1 average value	$Y_{1}$	$Y_{1}^{\prime }$	$\alpha_{1}$	$Z_{1}$	$\sigma_{1}^{\prime }$
B	1.07 average value	1.06 average value	$Y_{2}$	$Y_{2}^{\prime }$	$\alpha_{2}$	$Z_{2}$	$\sigma_{2}^{\prime }$
C	1.07 average value	1.14 average value	$Y_{3}$	$Y_{3}^{\prime }$	$\alpha_{3}$	$Z_{3}$	$\sigma_{3}^{\prime }$
D	1.06 average value	1.1 average value	$Y_{4}$	$Y_{4}^{\prime }$	$\alpha_{4}$	$Z_{4}$	$\sigma_{4}^{\prime }$
E	1.06 average value	1.06 average value	$Y_{5}$	$Y_{5}^{\prime }$	$\alpha_{5}$	$Z_{5}$	$\sigma_{5}^{\prime }$
F	1.06 average value	1.14 average value	$Y_{6}$	$Y_{6}^{\prime }$	$\alpha_{6}$	$Z_{6}$	$\sigma_{6}^{\prime }$
G	1.08 average value	1.1 average value	$Y_{7}$	$Y_{7}^{\prime }$	$\alpha_{7}$	$Z_{7}$	$\sigma_{7}^{\prime }$
H	1.08 average value	1.06 average value	$Y_{8}$	$Y_{8}^{\prime }$	$\alpha_{8}$	$Z_{8}$	$\sigma_{8}^{\prime }$
I	1.08 average value	1.14 average value	$Y_{9}$	$Y_{9}^{\prime }$	$\alpha_{9}$	$Z_{9}$	$\sigma_{9}^{\prime }$

Based on the above scenario setting and analysis, using the actual data of the new energy investment entity from 2015 to 2020, selecting key indicators, and further doing regression analysis, the annual investment scale forecast model is as follows:

$$Annual\;total\;investment=\left\{ \begin{array}{l} 0.916*consumer\;price\;index\\ +0.979\;*\;total\;profit\;\left(10000\;yuan\right) \end{array}\right.$$

Combined with the regression model, based on the design of the uncertainty multi-scenario simulation scheme, based on the average data from 2015 to 2019, the total investment scale in 2020 is predicted, and the results are shown below.

Combining with Table 10, it can be seen that, on the one hand, fully considering the influence of external factors and internal factors to construct a regression model of total investment, which can better predict the scale of total investment. Through deviation accounting, the overall deviation rate is within 2 %, which also indirectly verifies this paper. The total investment scale prediction model constructed from a macro perspective has certain applicability and scientificity. According to scenario A 1, it can be seen that when the resident consumption index and total profit are 1.07 times and 1.1 times the benchmark level respectively, the predicted investment is still 0.06 % less than the actual one. Therefore, when forecasting the investment scale, it is necessary to fully consider a variety of factors to make accurate calculations.

**Table 10:** Forecast of total investment under multi-scenario growth in 2020 based on average data from 2015 to 2019.

Scene	Predicted Residential Consumption Index (10,000 yuan)	Predicted Total profit	Forecast total input change (10,000 yuan)	Actual total investment (10,000 yuan)	Deviation
A	109.568	8.3204	108.5099596	108.572	−0.06 %
B	109.568	8.01784	108.2137534	108.572	−0.33 %
C	109.568	8.62296	108.8061658	108.572	0.22 %
D	108.544	8.3204	107.5719756	108.572	−0.92 %
E	108.544	8.01784	107.2757694	108.572	−1.19 %
F	108.544	8.62296	107.8681818	108.572	−0.65 %
G	110.592	8.3204	109.4479436	108.572	0.81 %
H	110.592	8.01784	109.1517374	108.572	0.53 %
I	110.592	8.62296	109.7441498	108.572	1.08 %

### 4.2 Results of differentiated new energy project priority evaluation

Based on the investment subjects of new energy projects selected in this paper, combined with the characteristics of investment projects, the qualitative indicators in the priority evaluation indicators are scored by experts, as shown in Table 11 below.

**Table 11:** Basic data for comprehensive evaluation of different new energy projects.

index	electric vehicle business	energy information service business	comprehensive energy business (coal-to-electricity business),	distributed energy business
A11	0.85	0.5	0.9	0.1
A12	0.8	0.5	0.85	0.05
A13	0.9	0.5	0.85	0.35
A14	90	90	90	85
A15	0.85	0.5	0.85	0.05
A21	95	85	95	80
A22	1	1	1	1
B11	8	9.5	8	8.5
B12	80	90	80	85
B13	80	90	60	85
B21	0.6	0.5	0.45	0.7
B22	14	12	14	16
B31	80	60	90	20
B32	80	50	90	85
C11	8	6	9.5	4
C12	8	6	9	2
C13	0.8	0.7	0.9	0.8
C14	50	50	90	30
C21	4	7.5	9.5	6
C22	90	80	80	90
C23	0.4	0.60	0.95	0.3
C31	80	90	90	85
C32	80	90	70	85

Based on Table 12, combined with the AN-EWM indicator weight determination method, the weight results are as follows.

**Table 12:** The most weight table of comprehensive evaluation index system for clean energy projects.

index	index	Weight of AN-EWM
A	A11	0.12596
	A12	0.18
	A13	0.051
	A14	0.00024
	A15	0.184
	A21	0.002
	A22	0.0013
B	B11	0.0022
	B12	0.001
	B13	0.009
	B21	0.015
	B22	0.0037
	B31	0.083
	B32	0.018
C	C11	0.04
	C12	0.09
	C13	0.0031
	C14	0.062
	C21	0.038
	C22	0.0014
	C23	0.085
	C31	0.001
	C32	0.0031

**Fig. 1:**
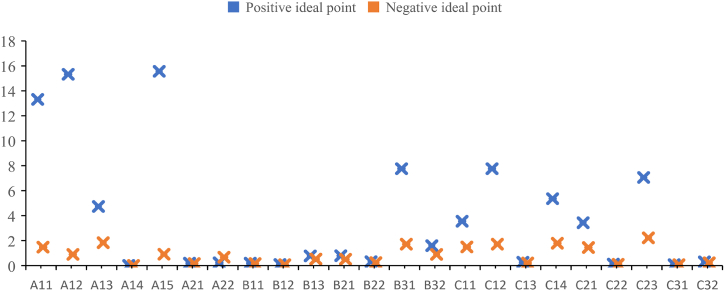
Positive and negative ideal points of TOPSIS for different new energy projects.

Based on the weight results, the subjective and objective weighting methods are used to weight the indexes of power grid projects. Then TOPSIS method is introduced to comprehensively evaluate the projects. The positive and negative ideal points of the power grid project can be determined by models (13)–(20), as shown in Fig. 1.

Further based on the model, the queuing indication value of each scheme can be calculated, as shown in Table 13.

**Table 13:** Results of prioritization of different new energy projects.

	electric vehicle business	energy information service business	comprehensive energy business (coal-to-electricity business)	distributed energy business
Distance from positive ideal point	29.55661399	148.9954991	0.22507659	686.0459958
Distance from negative ideal point	587.14915	201.84418	687.45619	1.05267
Queue indication value	0.952073	0.575317	0.999673	0.001532
Comprehensive sorting number	2	3	1	4

It can be seen from Table 13, first, combining with the TOPSIS model can realize the prioritization of different new energy projects and achieve precise investment. Secondly, it can be seen that thanks to the good social and economic benefits of the coal-to-electricity business, it can bring a relatively large investment recovery, and the technology is mature. The coal-to-electricity business is the first investment project of the new energy investor. Next, they are the electric vehicle business with good promotion prospects, the widely used information service business, and finally the distributed energy investment project with weak economic benefits.

## 5 Conclusion

This paper constructs an annual investment regression prediction model and a comprehensive evaluation model of differentiated project priorities from macro and micro perspectives for new energy project investors, which can effectively promote scientific and precise investment in new energy projects, and promote the scientific feasibility of new energy projects, the research conclusions are as follows:

Firstly, the multivariate regression annual total investment forecast model constructed in this paper from a macro perspective can predict the total investment scale. From the perspective of a complex model, the deviation rate of the annual total investment forecast can be kept within 5 % by comprehensively considering external and internal factors. From the perspective of sensitivity analysis, it can be seen that changes in load demand have an impact on the total investment scale. The factor is relatively large. When making investment, it is necessary to fully consider the change of load demand and make effective investment according to the demand. From the perspective of uncertainty changes, the regression model considering simple indicators can predict the total annual investment, which verifies that this paper is from a macro perspective. It is scientific to construct an impact index system, but the increase of positively correlated influencing factors does not necessarily lead to an increase in the total investment scale, and multi-factor changes should be fully considered.

Secondly, the investment priority models of different projects constructed in this paper from a micro perspective can realize the sorting of investment priorities. The priority comprehensive evaluation model proposed in this paper can effectively compare and evaluate different new energy projects from multiple perspectives such as economy and environment, realize the precise investment of new energy project investors, and further promote new energy while improving social benefits. The scientific development of the project investor itself.

Investment scale prediction is an important part of power system development of clean energy project. Strengthening the prediction of investment prediction can make companies better allocate assets and improve investment management level and investment efficiency. Therefore, in the development process of clean energy project, we must constantly improve the investment forecasting technology, realize accurate investment, and maximize the economic and social benefits of the company's development.

## Data availability statement

In response to question one, the significance of the research in this paper and the gaps it fills are further highlighted. In response to question two, the article includes the process of data collation and the software used (including its version). In response to question six, this paper adds a Chinese context to the article and also improves the abstract section.

## Additional information

No additional information is available for this paper.

## CRediT authorship contribution statement

**Guang Tian:** Writing - original draft, Resources. **Xiangyu Chen:** Writing - original draft, Data curation. **Chunsheng Chen:** Writing - original draft, Conceptualization. **Yang Yang:** Writing - original draft. **Jialin Li:** Conceptualization. **Yangyi Zhang:** W.

## Declaration of competing interest

The authors declare that they have no known competing financial interests or personal relationships that could have appeared to influence the work reported in this paper.
